# Propensity Matched Outcomes of Minimally Invasive Mitral Surgery: Does a Heart-Team Approach Eliminate Female Gender as an Independent Risk Factor?

**DOI:** 10.3390/jpm13060949

**Published:** 2023-06-03

**Authors:** Laina Passos, Isabel Lavanchy, Thierry Aymard, Mohammed Morjan, Ioannis Kapos, Roberto Corti, Juerg Gruenenfelder, Patric Biaggi, Diana Reser

**Affiliations:** 1 Heart Clinic Hirslanden, Witellikerstrasse 40, 8032 Zuerich, Switzerland; 2 Department of Cardiac Surgery, Medical Faculty, University Hospital Duesseldorf, Heinrich-Heine-University Duesseldorf, Mooren Str. 5, 40225 Duesseldorf, Germany

**Keywords:** heart team, minimally invasive mitral surgery, female gender an independent risk factor

## Abstract

Background: There is increasing evidence that female gender is an independent risk factor for cardiac surgery. Minimally invasive mitral surgery (MIV) has proven to have excellent long-term results, but little is known about gender-dependent outcomes. The aim of our study was to analyze our heart team's decision-based MIV-specialized cohort. Methods: In-hospital and follow-up data were retrospectively collected. The cohort was divided into gender groups and propensity-matched groups. Results: Between 22 July 2013 and 31 December 2022, 302 consecutive patients underwent MIV. Before matching, the total cohort showed that women were older, had a higher EuroSCORE II, were more symptomatic, and had more complex valve pathology and tricuspid regurgitation resulting in more valve replacements and tricuspid repairs. Intensive and hospital stays were longer. In-hospital deaths (n = 3, all women) were comparable, with more atrial fibrillation in women. The median follow-up time was 3.44 (0.008–8.9) years. The ejection fraction, NYHA, and recurrent regurgitation were low and comparable and atrial fibrillation more frequent in women. The calculated 5-year survival and freedom from re-intervention were comparable (*p* = 0.9 and *p* = 0.2). Propensity matching compared 101 well-balanced pairs; women still had fewer resections and more atrial fibrillation. During the follow-up, women had a better ejection fraction. The calculated 5-year survival and freedom from re-intervention were comparable (*p* = 0.3 and *p* = 0.3). Conclusions: Despite women being older and sicker, with more complex valve pathology and subsequent replacement, early and mid-term mortality and the need for reoperation were low and comparable before and after propensity matching, which might be the result of the MIV setting combined with our patient-tailored decision-making. We believe that a multidisciplinary heart team approach is crucial to optimize patient outcomes in MIV, and it might also reduce the widely reported increased surgical risk in female patients. Further studies are needed to prove our findings.

## 1. Introduction

There is increasing evidence that female patients have worse early and late outcomes in coronary, aortic and valve surgeries [1,2,3,4]. The reasons for this might be the following: women present with atypical symptoms (leading to under-diagnosis or misdiagnosis), are referred later for surgery (presenting older and with more comorbidities) and less often (despite a higher incidence of mitral prolapse) due to guidelines, which are derived from research mainly performed on male subjects [1,5,6,7]. Furthermore, women more often receive mitral valve replacement, and mitral repair does not restore their normal life expectancy [8]. Therefore, the female gender has been identified as an independent risk factor for mortality after cardiac surgery and was included in the risk assessment scores (EuroSCORE II and STS Score) [9,10]. Minimally invasive video-assisted mitral valve surgery (MIV) is the standard of care at specialized centers worldwide, shows excellent short and long-term outcomes and is even superior to sternotomy, as shown in meta-analyses and propensity-matched studies [11,12,13,14]. However, little is known about gender-dependent outcomes in MIV. The EACTS guidelines suggest the “heart team” as a class 1C indication, which makes it a mandatory decision-making organ in the presence of an increasing number of transcatheter treatment options [15]. However, there is still a lack of evidence regarding the influence of heart-team decision-making on gender-dependent outcomes. Therefore, the aim of our study was to analyze the outcomes of our single-center heart team’s decision-based and MIV-specialized cohort with regard to gender.

## 2. Materials and Methods

In 2013, we established a structural heart team center where every single valve case is discussed in an interdisciplinary approach to enable patient-tailored therapies (cardiac surgery, percutaneous, or conservative treatment) and, consequently, to reduce interventional risk. The concept and preliminary outcomes of our heart team approach comparing the outcomes of MIV and Mitra-clip patients have already been published elsewhere [16]. Based on this decision-making by our institutionalized multidisciplinary heart team, we aimed to analyze our MIV cohort according to gender-related outcomes: preoperative characteristics, in-hospital outcomes (30 days) and follow-up information (survival, valve competence, freedom from reoperation) were collected and analyzed retrospectively from our institutional database, from follow-up records of our referring cardiologists, and prospectively by contacting all patients by phone. Ethics committee approval from our local institutional review board (amendment to the approval number 2017-01895) and signed informed consent were obtained from every patient in this study. Our heart team meets weekly and consists of cardiac surgeons, cardiologists (interventionalist and non-invasive cardiologists, including imaging specialists), cardiac anesthetists and intensivists. It is a financially independent organ, where every valve case is discussed and decision-making is performed according to guidelines and surgical risk assessments: patients younger than 80 years without excessive operative risk (i.e., EuroSCORE II < 4%) are primarily evaluated for MIV feasibility, “high risk” patients and those with a morphological lower probability for successful surgical MIV-repair are treated percutaneously with edge-to-edge technique (MitraClip, Abbott or Pascal Edwards). We are a specialized MIV center and our only contraindications for this approach are severe coronary disease requiring grafting with ischemic regurgitation, suspected adhesions in the right thorax (previous surgery or irradiation), severe annular calcification and grade III-IV arteriosclerosis of the ascending and/or descending aorta.

**Figure 1:**
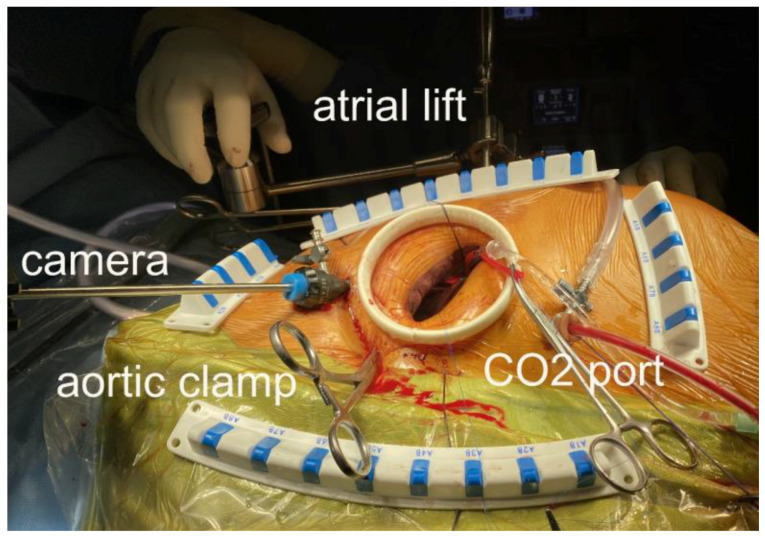
Video-assisted minimally invasive mitral valve surgery: intraoperative setting.

Our surgical technique has already been described previously [17]; femoral cannulation is performed either percutaneously or surgically (Seldinger technique). Figure 1 shows the intraoperative settings. CO_2_ insufflation through a caudal port is mandatory. A long aortic root cannula is used for antegrade cardioplegia (Custodiol, Brettschneider), which is also used as a vent for de-airing. The atrial lift retractor is inserted through a right parasternal hole under camera guidance in order to avoid injuring the mammary artery. We implant an annuloplasty ring in all repair cases. When resection is required (excess tissue), we perform it triangularly (small area to avoid tension and immobility). When neo-chordae are required, we use single Goretex sutures (Gore, Newark, DE, USA). Further repair techniques include cleft closures, commissuroplasty and very rarely, sliding plasty or Alfieri-Stich. Concomitant procedures include left atrial appendage closure with sutures or clips, cryoablation, patent foramen ovale or ASD closure and tricuspid valve annuloplasty when indicated. During the mitral valve replacement, we resect the anterior leaflet only. Mitral repair and biological prosthesis patients receive warfarin for 3 months (mechanical valves are lifelong). All patients receive an echocardiogram by our specialized interventional imaging cardiologists in the theatre after de-clamping (transesophageal) and before discharge (transthoracic) to evaluate the results of the surgery. Regurgitation grade is defined from 1 to 4 with none and trace, mild, moderate, or severe. After the rehabilitation period, patients are evaluated clinically and echocardiographically by their referring cardiologists initially after 3 months, then after 1 year and annually thereafter.

All statistical analyses were performed using R version 4.2.1 (The R Foundation for Statistical Computing, Vienna, Austria). Categorical variables are presented as frequencies with percentages and compared between groups using Fisher’s exact test. Continuous variables are presented as mean ± standard deviation (SD) and compared between groups using the Mann–Whitney test. Overall survival and freedom from re-intervention are presented as Kaplan–Meier curves and compared between groups using the log-rank test. Propensity score matching was used to overcome the inherent imbalance between female and male patients. Using logistic regression, the propensity score (PS) for the probability of being female was calculated for each operated patient. The used covariates in the model were EuroSCORE II (without female regression coefficient of 0.2196434), age, ejection fraction, atrial fibrillation, NYHA III/IV, COPD, coronary artery disease (CAD), previous stroke, previous percutaneous coronary intervention (PCI), Barlow’s disease, single-segment pathology, two-segment pathology anterior or posterior, posterior pathology only, anterior and posterior pathology without Barlow’s, commissural pathology, leaflet calcification and severe tricuspid regurgitation. We performed one-to-one nearest neighbour propensity score matching without replacement with a caliper of 0.2 of the SD of the logit-transformed PS. After matching, all standardized mean differences (SMD) for the covariates were below 0.1, indicating an adequate balance [18].

Due to the small number of events, multivariable analysis, including possible variable transformations, interactions, cut-off values and model checking, would not be the correct method of analysis. Hence, we decided not to perform it in our study.

## 3. Results

Between 22 July 2013 and 31 December 2022 a total of 302 consecutive elective patients underwent MIV at our clinic performed by four surgeons (during the same period, our institutional cardiologists treated 349 patients percutaneously with edge-to-edge repair). In order to compare gender-dependent outcomes, we defined two groups: male and female.

### 3.1. Total Unmatched Cohort

Table 1 shows the baseline characteristics; majority of the participants were men (58.6%). The EuroSCORE II, age, the incidence of NYHA III/IV, previous PCI, Barlow’s, leaflet pathology, annular calcification and tricuspid regurgitation were significantly different between the groups. 

Table 2 shows the in-hospital outcomes; cross-clamp and bypass times were comparable between the groups (*p* = 0.28 and 0.34). There was only one conversion to clamshell in the entire cohort, and there was no need for conversion to sternotomy. Women had significantly more planned and performed valve replacements (*p* = 0.0002), despite a comparable rate of intraoperatively failed repair (*p* = 0.39). Men had more leaflet resections (*p* < 0.001), with similar incidences of further repair techniques and concomitant procedures. Women had significantly more additional tricuspid valve repairs than men (*p* < 0.001). Intensive unit and hospital stays were longer for women (*p* = 0.05 and *p* = 0.01). There were three in-hospital deaths (0.99%), all of whom were women; two were due to cardiogenic shocks despite an ECMO implant (on post-operative days three and five) and one was due to severe pulmonary edema on day 30. However, mortality did not show a significant difference compared to men (*p* = 0.07). Stroke, rethoracotomy, ECMO-implant and need for re-operation were comparable between the groups. The incidence of postoperative atrial fibrillation was higher in women (*p* = 0.003). Only one patient needed in-hospital re-operation due to SAM on day nine (which was successfully repaired).

Table 3 shows the follow-up outcomes: median follow-up was 3.44 years (0.008–8.9 years and interquartile range of 1.59 to 5.33 years). The well-balanced distribution of the follow-up of the total unmatched cohort is shown in the upper part of the supplementary histogram (Figure S1). Only five patients (1.7%, all men) refused to participate in the follow-up and four were lost to follow-up due to moving abroad (1.3%, all men). Follow-up of the 299 survivors (99%) was completed in 247 patients (85.6%) by our referring cardiologists until 31 December 2022 (up to 9.5 years). At that time, another 43 patients (14.4%) had a pending follow-up appointment but were found to be alive and well after contacting them by phone. We closed the follow-up period on 31 December 2022. At discharge, 100% of the patients completed echocardiographic follow-up after 3 months 85%, at 1 year 68% and thereafter 89%. NYHA and the incidence of recurrent mitral regurgitation were stable, constant over time and comparable between the groups. Mitral regurgitation of less than grade 2 could be confirmed in 96.6% of the followed-up patients. There was no relevant mitral stenosis, but the gradient was significantly higher in women beyond 1 year (*p* = 0.03). The incidence of atrial fibrillation was significantly higher in women after three months (19.7% vs. 10.6%, *p* = 0.01) and 1 year (11.5% vs. 4.9%, *p* = 0.03) and decreased over time. The ejection fraction and tricuspid regurgitation were higher in women beyond 1 year (*p*= 0.005 and *p* = 0.04, respectively).

**Figure 2:**
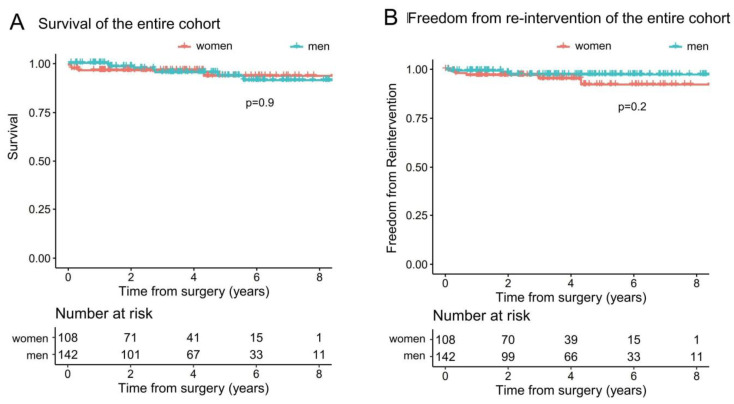
Kaplan–Meier curves of the unmatched total cohort. (**A**) Women had a 1-year survival of 96.2% (95% CI 92.7–99.9%) and 5-year survival of 93.4% (95% CI 87–100%). Men had a 1-year survival of 100% and 5-year survival of 93.5% (95% CI 88–98%). (**B**) Women had a 1-year freedom from re-intervention of 97% (95% CI 93.8–100%) and 5-year of 92.2% (95% CI 85–100%). Men had a 1-year freedom from re-intervention of 99.3% (95% CI 97.9–100%) and 5-year of 97.3% (95% CI 94.3–100%).

Figure 2A shows the calculated survival rates, which were comparable (*p* = 0.9). Women had a 1-year survival of 96.2% (95% CI 92.7–99.9%) and 5-year survival of 93.4% (95% CI 87–100%). Men had a 1-year survival of 100% and 5-year survival of 93.5% (95% CI 88–98%). There were nine late deaths (3%), including two women and seven men (*p* = 0.3): one was due to cardiac, four non-cardiac and four unknown causes. There was no cerebrovascular event or stroke. 

Figure 2B shows freedom from re-intervention, which was comparable (*p* = 0.2). Women had a 1-year freedom from re-intervention of 97% (95% CI 93.8–100%) and 5-year of 92.2% (95% CI 85–100%). Men had a 1-year freedom from re-intervention of 99.3% (95% CI 97.9–100%) and 5-year of 97.3% (95% CI 94.3–100%). Eight patients (2.7%) needed re-intervention for recurrence of mitral regurgitation grade > 3; one due to SAM, two due to endocarditis and five due to recurrent prolapse. Two received a Mitra-Clip, and one could be re-repaired. These outcomes were also comparable between the groups.

### 3.2. Propensity Score Matched Groups

**Figure 3:**
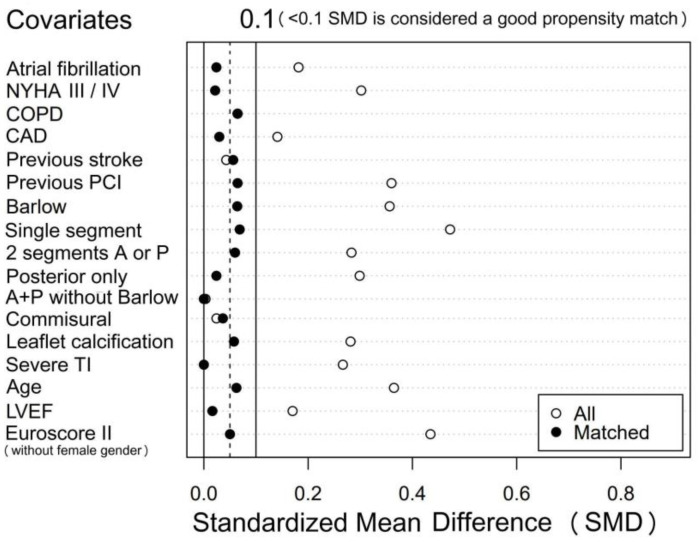
Propensity score matching for both genders: standardized mean differences (SMD) of the unmatched (white dots) and matched (black dots) groups. All dots below 0.1 SMD were considered a good match. We deducted the female regression coefficient of 0.2196434 included in the EuroSCORE II in order to be able to use it for propensity matching. NYHA = New York Heart Association functional class; COPD = chronic obstructive pulmonary disease; CAD = coronary artery disease; PCI = percutaneous coronary intervention; A = anterior leaflet; P = posterior leaflet; LVEF = left ventricular ejection fraction.

From the entire cohort of 302 patients, our propensity score matched 202 (101 in each group, 81% of the female patients with 57% of the male patients). Further matching was not possible because of the lack of age matching for the remaining elderly women. Figure 3 shows a well-balanced match of the covariates used, with all standardized mean differences (SMD) less than 0.1.

Table 4 shows the baseline characteristics of the matched gender groups; the previously mentioned significant differences between the unmatched groups could not be detected anymore.

Table 5 shows the in-hospital outcomes of the matched gender groups; only leaflet resection and atrial fibrillation showed a significant difference. Women still had fewer leaflet resections (*p* = 0.001) and more atrial fibrillation (*p* = 0.04) than men.

Table 6 shows the follow-up outcomes of the matched gender groups; the median follow-up was 3.44 (0.008–8.7 years with an interquartile range of 1.45 to 5.25 years) and is comparable to the total cohort. The well-balanced distribution of the follow-up of the matched gender groups is shown in the lower part of the supplementary histogram (Figure S1). The only significant difference was a better ejection fraction in women after 1 year of follow-up (*p* = 0.04) and beyond (*p* = 0.004), as well as a slightly higher mitral gradient beyond 1 year (*p* = 0.05).

**Figure 4:**
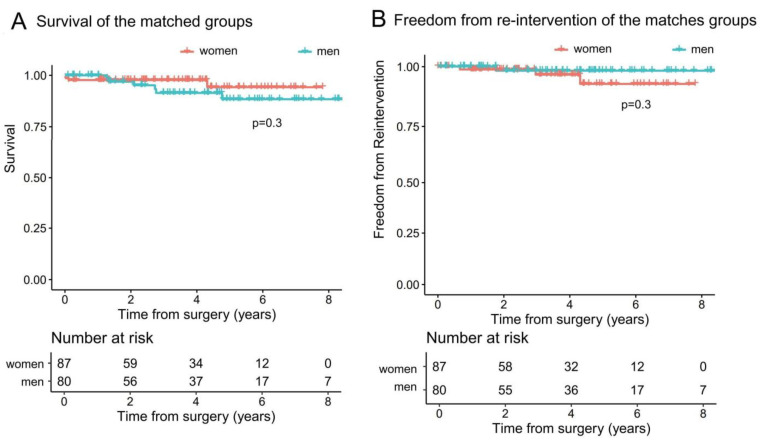
Kaplan–Meier curves of the matched groups. (**A**) Women had a 1-year survival of 97.7% (95% CI 94.6–100%) and 5-year survival of 94.2% (95% CI 87.1–100%). Men had a 1-year survival of 100% and a 5-year survival of 88.4.5% (95% CI 79.7–98%). (**B**) Women had a 1-year freedom from re-intervention of 98.8% (95% CI 96.3–100%) and a 5-year of 92.7% (95% CI 84.3–100%). Men had a 1-year freedom from re-intervention of 98.2% (95% CI 94.8–100%).

Figure 4A shows the calculated survival of the matched gender groups, which were comparable (*p* = 0.3). Women had a 1-year survival of 97.7% (95% CI 94.6–100%) and 5-year survival of 94.2% (95% CI 87.1–100%). Men had a 1-year survival of 100% and 5-year survival of 88.4.5% (95% CI 79.7–98%).

Figure 4B shows freedom from re-intervention of the matched gender groups, which were also comparable (*p* = 0.3). Women had a 1-year freedom from re-intervention of 98.8% (95% CI 96.3–100%) and 5-year of 92.7% (95% CI 84.3–100%). Men had a 1-year freedom from re-intervention of 100%, 95% CI 100–100% and 5-year of 98.2% (95% CI 94.8–100%).

## 4. Discussion

Our data from a multidisciplinary structural heart team decision-based MIV cohort suggest, that despite women being older and sicker, with more complex valve pathology and subsequent replacement, more tricuspid repair, longer ICU and hospital stay and more atrial fibrillation, early and mid-term mortality, morbidity and re-intervention are still low and comparable to those of men. We believe that these favorable outcomes might be the combined result of our heart team approach, the MIV setting and the experienced surgeons, which seems to eliminate the female gender as an independent risk factor for cardiac surgery.

It could be argued that another reason for our good results might be the low-risk patient selection for MIV because the majority of degenerative mitral cases usually present with a single segment P2 prolapse, which can be easily repaired, resulting in excellent durability and a life expectancy comparable to the general population in both MIV and sternotomy studies [19,20,21,22]. However, our findings prove otherwise: despite a high incidence of mixed and complex valve pathologies in our MIV cohort (predominantly in women), we have a low mid-term morbidity/mortality and re-intervention rate, which is comparable to the outcomes in men. In our preliminary study of our heart team's decision-based approach, we showed that women older than 75 years and smaller than 170 cm have an increased risk if they are treated with MIV (16). With this finding in mind and the outcomes of our present analysis, it seems that our heart team approach was able to eliminate the female gender as an independent risk factor for MIV. Several studies of sternotomy cohorts showed that anterior and/or bi-leaflet pathologies, tissue thickening and calcifications are more common in women (comparable to our findings); therefore, they are more complex to repair with longer cross-clamping, which results in higher morbidity and mortality and an increased risk for recurrent mitral regurgitation in women [19,21,23,24]. A large study of Medicare beneficiaries (n = 183,792) also revealed a significantly increased early mortality after mitral surgery through sternotomy in women but comparable long-term outcomes [8]. Furthermore, it was shown that women received mitral valve replacement more often due to more complex valve pathologies, and mitral repair only restored normal life expectancy in men but not in women [8]. Similarly, Johnston et al. found that female patients had comparable early mortality and better survival after mitral valve replacement than men, whereas men had better survival after mitral repair [3]. Propensity-matched studies comparing MIV versus sternotomy showed that women were older at admission and less likely to be considered for MIV, but with comparable results for both gender groups and surgical techniques [25,26]. When comparing the outcomes of surgical and percutaneous mitral valve treatments in the elderly, it was shown that there was lower morbidity and mortality up to 1 year compared with surgery, but a higher recurrence of regurgitation and mortality beyond [27,28]. 

In the past decades, selective studies have made us aware of the fact that, despite women having a higher incidence of mitral prolapse, fewer are referred for surgery, and they are older, sicker, have lower repair rates and worse outcomes than men after cardiac surgery through sternotomy [1,2,4,5,7,29,30,31]. The reasons for this might be misdiagnosis (mild and/or atypical cardiovascular symptoms) and underdiagnosis (women less often reach diameter-based surgical guideline criteria because they were originally established for men) [1,5,6,7,29,31,32]. A propensity-matched report of 270 gender pairs showed comparable gender outcomes through sternotomy due to their dedicated use of speckle-tracking echo analysis, which can reveal ventricular dysfunction and prevent delayed surgery in women [33]. We did not use speckle-tracking, and in our cohort, women were also older and sicker (which could be a sign of delayed referral). However, short- and mid-term mortality and the need for re-intervention were low and comparable to those in men.

We believe that our favorable outcomes are the result of our multidisciplinary heart team decision-making concept, which allows patient-tailored medicine combined with the dedicated work of our experienced surgeons and the MIV approach itself, which has proven to be advantageous mainly in high-risk and elderly patients [34]. However, the Leipzig group also showed in their large all-comer MIV cohort that the female gender is an independent predictor of cardiac mortality with significantly worse long-term survival in women. They had less posterior leaflet pathology and received more valve replacements and tricuspid surgery than men, which is comparable to our findings [19,22]. We still have to wait for our long-term data in order to confirm our excellent and comparable mid-term outcomes, but we are confident that the re-intervention rate will remain low because the incidence mainly occurs at a mean of 15 months [35].

Our propensity-matched analysis showed a 5-year survival of women with 94.2% (95% CI 87.1–100%) and men with 88.4.5% (95% CI 79.7–98%) (*p* = 0.03), which is better than that reported in other studies (65–85%), where anterior and bi-leaflet pathologies showed the worst outcomes [19,23,36,37]. Our freedom from re-intervention at 5 years is 92.7% (95% CI 84.3–100%) in women and 98.2% (95% CI 94.8–100%) in men (*p* = 0.03) and is comparable or better than in other studies (90–96%) where anterior leaflet pathology is described as the only independent predictor of reoperation [19,22,23,36]. At the closing of the follow-up on 31 December 2022 (up to 9.5 years), only two patients (0.7%, both women) had New York Heart Association function class III/IV symptoms and 96.3% of the valves were competent (<grade 2 regurgitation), which was constant over time (comparable between the groups). These findings are similar or superior to those described in other studies with up to 11% NYHA III/IV in long-term follow-up [20,23].

The limitations of our study are the following: it is a low-volume, single-center, retrospective observational study, which allows for potential biases. There was no control group available because we did not perform median sternotomy for isolated mitral valve surgery at our institution. Furthermore, we decided not to include our Mitra-Clip cohort again, since we already published a comparison of the two cohorts previously. The strength of this study is the use of propensity score matching. There is a variation in the follow-up duration (a few weeks up to 9 years), which reduces the validity of the long-term results. We are a specialized heart team decision-based MIV center and our outcomes might not be reproducible elsewhere. Our MIV patient cohort was low-risk, and some were lost to follow-up or were waiting for outpatient visits. Except for in-hospital echocardiography, we did not use a core lab for follow-up imaging, which might have resulted in interpretational bias. 

## 5. Conclusions

Despite our findings that women are older and sicker, with more complex valve pathology and subsequent replacement, early- and mid-term mortality and the need for reoperation were low and did not differ between the gender groups before and after propensity matching, which might be the result of the MIV setting combined with our patient-tailored decision-making. We believe that a multidisciplinary heart team approach is crucial to optimize patient outcomes in MIV and it might also reduce the widely reported increased surgical risk of female patients. However, further studies are needed to prove these findings.

## Figures and Tables

**Table 1:** Baseline characteristics of the total cohort according to gender.

	Male	Female	*p*
Number of patients	177 (58.6)	125 (41.4)	-
EuroSCORE II (%)	1.23 ± 0.98	1.98 ± 1.35	<0.001
Age (years)	62.6 ± 11.9	66.8 ± 11.6	<0.001
LVEF (%)	62.9 ± 7.41	63.9 ± 6.52	0.38
NYHA Class (III/IV)	28 (15.8)	37 (29.6)	0.005
Atrial fibrillation	25 (14.1)	27 (21.6)	0.12
Previous stroke	7 (4)	4 (3.2)	1
Coronary heart disease	31 (17.5)	16 (12.8)	0.33
Previous PCI	14 (7.9)	3 (2.4)	0.045
COPD	3 (1.7)	6 (4.8)	0.74
Hypertension	51 (28.8)	38 (30.4)	0.8
Peripheral vascular disease	3 (1.7)	2 (1.6)	1
Previous cardiac surgery	2 (1.1)	1 (0.8)	1
Mitral regurgitation	175 (98.9)	119 (95.2)	0.07
Mitral stenosis	2 (1.1)	6 (4.8)	0.07
Degenerative mitral disease	174 (98.3)	118 (94.4)	0.1
Functional mitral disease	1 (0.6)	6 (4.8)	0.02
Barlow’s disease	26 (14.7)	39 (31.2)	<0.001
Single-segment pathology	80 (45.2)	31 (24.8)	<0.001
Double-segment pathology	53 (30)	55 (55)	0.015
Posterior leaflet pathology	60 (33.9)	27 (21.6)	0.02
Antero-posterior pathology	38 (21.5)	27 (21.6)	1
Commissural pathology	13 (7.3)	10 (8)	0.83
Flial leaflet	98 (55.4)	34 (27.2)	<0.001
Leaflet calcification	7 (4)	17 (13.6)	0.004
Annular calcification	18 (10.2)	26 (20.8)	0.02
Tricuspid valve regurgitation			
Moderate	10 (5.6)	16 (12.8)	0.038
Severe	4 (2.3)	13 (10.4)	0.004

LVEF = left ventricular ejection fraction; NYHA = New York Heart Association; PCI = percutaneous coronary intervention; COPD = chronic obstructive pulmonary disease.

**Table 2:** In-hospital outcomes of the total cohort according to gender.

	Male	Female	*p*
Number of patients	177 (58.6)	125 (41.4)	-
Bypass time (minutes)	164 ± 47	160 ± 46	0.34
Cross-clamp time (minutes)	107 ± 36	103 ± 32	0.28
Conversion (to clamshell)	0	1 (0.8)	0.41
Successful valve repair	164 (92.6)	97 (77.6)	<0.001
- Ring size in millimetre	34.8 ± 2.2	33.6 ± 2.8	<0.001
Leaflet resection	81 (45.7)	26 (20.8)	<0.001
Neochordae	60 (33.9)	30 (24)	0.07
Sliding plasty	11 (6.2)	3 (2.4)	0.16
Cleft closure	82 (46.3)	55 (44)	0.72
Commissuroplasty	14 (7.9)	15 (12)	0.24
Baleout replacement	6 (3.4)	7 (5.6)	0.39
Valve replacement	13 (7.4)	28 (22.4)	<0.001
- mechanical	0	3 (2.4)	0.07
- biological	19 (10.7)	33 (26.4)	<0.001
- size in millimetre	30.6 ± 1.8	29.2 ± 1.6	0.02
Tricuspid valve repair	8 (4.5)	28 (22.4)	<0.001
Cryoablation	5 (2.8)	7 (5.6)	0.24
Left atrial appendage closure			
- Clip	9 (5.1)	12 (9.6)	0.16
- Suture	11 (6.2)	14 (11.2)	0.14
Foramen ovale closure	16 (9)	16 (12.8)	0.34
In-hospital mortality	0	3 (2.4)	0.06
Permanent stroke	4 (2.3)	1 (0.8)	0.4
Tamponade	0	1 (0.8)	0.4
Rethoracotomy	1 (0.6)	3 (2.4)	0.3
Intensive care stay (days)	1.8 ± 2.8	2.2 ± 3.6	0.05
Hospital stay (days)	10.3 ± 4.4	11.2 ± 4.7	0.01
Pacemaker implantation	12 (6.8)	9 (7.2)	1
Post-op ECMO	1 (0.6)	2 (1.6)	0.5
Need for re-operation	1 (0.6)	0	0.5
LVEF postop (%)	56.3 ± 7.6	58.2 ± 8.1	0.12
Incidence of atrial fibrillation	18 (10.2)	29 23.3)	0.003
Mitral regurgitation > 2	6 (3.4)	2 (1.6)	0.48
Mitral gradient (mmHg)	3 ± 1.9	3.2 ± 1.5	0.29
Tricuspid regurgitation > 2	1 (0.6)	3 (2.4)	0.42

ECMO = extracorporeal membrane oxygenation; LVEF = left ventricular ejection fraction.

**Table 3:** Follow-up outcomes of the total cohort according to gender.

	Male	Female	*p*
Survivors	177 (100)	122 (97.5)	0.07
Patients followed up	142 (80.2)	105 (86.1)	0.17
Patients abroad (lost to FU)	4 (2.3)	0	0.14
Patients did not consent	5 (2.8)	0	0.08
Waiting for follow up	26 (14.7)	17 (13.9)	0.87
Stroke	0	0	-
Mortality	7	2	0.9
Reintervention	3	5	0.2
LVEF			
- After 3 months	57.6 ± 7.2	59.3 ± 7.3	0.06
- After 1 year	57.7 ± 7.8	60.8 ± 6.3	0.01
- >1 year	57.9 ± 8.2	61.8 ± 6.2	0.005
NYHA III/IV			
- 3 months	3 (2.1)	1 (0.8)	0.63
- 1 year	3 (2.1)	1 (0.8)	0.26
- >1 year	0	2 (1.6)	0.15
Atrial fibrillation			
- After 3 months	15 (10.6)	24 (19.7)	0.01
- After 1 year	7 (4.9)	14 (11.5)	0.03
- >1 year	7 (4.9)	11 (9.0)	0.07
Tricuspid regurtigation > grade 2			
- After 3 months	2 (1.4)	1 (0.8)	1
- After 1 years	2 (1.4)	3 (2.5)	0.65
- >1 year	0	4 (3.3)	0.04
Mitral regurgitation > grade 2			
- 3 months	6 (4.2)	4 (3.3)	1
- 1 year	6 (4.2)	4 (3.3)	1
- >1 year	7 (4.9)	4 (3.3)	0.82
Mitral gradient (mmHg)			
- 3 months	2.7 ± 1.4	2.9 ± 1.3	0.06
- 1 year	2.7 ± 1.4	2.9 ± 1.6	0.36
- >1 year	2.5 ± 1.2	3.2 ± 1.5	0.03

**Table 4:** Baseline characteristics after propensity score matching.

	Male	Female	*p*
Number of patients	101	101	-
EuroSCORE II (%)	1.4 ± 1.2	1.4 ± 0.9	0.46
Age (years)	65.1 ± 11.5	65.8 ± 12.1	0.5
LVEF (%)	64.1 ± 7.3	64.2 ± 5.8	0.6
NYHA Class (III/IV)	21 (20.8)	20 (19.8)	1
Atrial fibrillation	17 (16.8)	18 (17.8)	1
Previous stroke	5 (4.95)	4 (3.96)	1
Coronary heart disease	14 (13.9)	15 (14.9)	1
Previous PCI	1 (0.99)	2 (1.98)	1
COPD	4 (3.96)	3 (2.97)	1
Hypertension	30 (29.7)	32 (31.7)	0.9
Peripheral vascular disease	2 (1.98)	1 (0.99)	1
Previous cardiac surgery	2 (1.98)	1 (0.99)	1
Mitral regurgitation	99 (98)	98 (97)	1
Mitral stenosis	2 (1.98)	3 (2.97)	1
Degenerative mitral disease	99 (98)	98 (97)	1
Functional mitral disease	1 (0.99)	2 (1.98)	1
Barlow’s disease	24 (23.8)	27 (26.7)	0.7
Single-segment pathology	32 (31.7)	29 (28.7)	0.7
Double-segment pathology	38 (37.6)	41 (40.6)	0.8
Posterior leaflet pathology	27 (26.7)	26 (25.8)	1
Antero-posterior pathology	22 (21.8)	22 (21.8)	1
Commissural pathology	9 (8.9)	8 (7.9)	1
Leaflet calcification	6 (5.9)	8 (7.9)	0.8
Annular calcification	13 (12.9)	21 (20.8)	0.3
Tricuspid valve regurgitation			
Moderate	6 (5.9)	10 (9.9)	0.4
Severe	4 (3.96)	4 (3.96)	1

LVEF = left ventricular ejection fraction; NYHA = New York Heart Association; PCI = percutaneous coronary intervention; COPD = chronic obstructive pulmonary disease.

**Table 5:** In-hospital outcomes after matching.

	Male	Female	*p*
Number of patients	101	101	-
Bypass time (minutes)	167 ± 51	162 ± 47	0.35
Cross-clamp time (minutes)	108 ± 39	104 ± 34	0.35
Conversion (to clamshell)	0	1 (0.99)	1
Successful valve repair	91 (90.1)	84 (81.2)	0.21
- Ring size in millimetre	34.7 ± 2.1	33.6 ± 2.8	0.007
Leaflet resection	47 (46.5)	24 (23.8)	0.001
Neochordae	30 (29.7)	30 (29.7)	1
Sliding plasty	8 (7.9)	3 (2.97)	0.21
Cleft closure	45 (44.6)	49 (48.5)	0.67
Commissuroplasty	5 (4.95)	15 (14.9)	0.03
Baleout replacement	4 (3.96)	7 (6.93)	0.53
Valve replacement	10 (9.9)	17 (16.8)	0.21
- mechanical	0	2 (1.98)	0.49
- biological	14 (13.9)	22 (21.8)	0.19
- size in millimetre	30.9 ± 1.9	29 ± 1.6	0.009
Tricuspid valve repair	6 (5.94)	15 (14.9)	0.06
Cryoablation	3 (2.97)	2 (1.98)	1
Left atrial appendage closure			
- Clip	7 (6.93)	9 (8.9)	0.79
- Suture	4 (3.96)	8 (7.9)	0.37
Foramen ovale closure	7 (6.93)	10 (9.9)	0.61
In-hospital mortality	0	2 (1.98)	0.49
Permanent stroke	3 (2.97)	1 (0.99)	0.62
Tamponade	0	1 (0.99)	1
Rethoracotomy	1 (0.99)	1 (0.99)	1
Intensive care stay (days)	2 ± 3.6	1.8 ± 2	0.39
Hospital stay (days)	10.9 ± 5.5	10.6 ± 3.3	0.5
Pacemaker implantation	8 (7.92)	5 (4.95)	0.56
Post-op ECMO	1 (0.99)	2 (1.98)	1
Need for re-operation	0	0	-
LVEF postop (%)	57.5 ± 7.5	57.3 ± 7.9	0.67
Incidence of atrial fibrillation	10 (9.9)	21 (20.8)	0.04
Mitral regurgitation > 2	3 (2.97)	1 (0.99)	0.49
Mitral gradient (mmHg)	3 ± 1.4	3.3 ± 1.5	0.23
Tricuspid regurgitation > 2	0	3 (2.97)	0.18

ECMO = extracorporeal membrane oxygenation; LVEF = left ventricular ejection fraction.

**Table 6:** Follow-up outcomes after matching.

	Male	Female	*p*
Survivors	101	99	0.5
Patients followed up	80 (79.2)	87 (87.9)	0.26
Patients abroad (no FU)	1 (0.99)	0	1
Patients did not consent	3 (2.97)	0	0.25
Waiting for follow up	17 (16.8)	14 (14.14)	0.67
Stroke	0	0	-
Mortality	6 (5.9)	2 (2.1)	0.3
Reintervention	1 (0.99)	3 (3.45)	0.3
LVEF			
- After 3 months	57.9 ± 7.3	59.8 ± 7.0	0.1
- After 1 year	57.8 ± 6.8	60.8 ± 6.0	0.04
- >1 year	58.1 ± 7.9	62.6 ± 5.8	0.004
NYHA III/IV			
- 3 months	1 (1.25)	0	0.48
- 1 year	0	2 (2.3)	0.22
- >1 year	0	2 (2.3)	0.49
Atrial fibrillation			
- After 3 months	10 (12.5)	13 (15)	0.66
- After 1 year	6 (7.5)	11 (12.6)	0.43
- >1 year	5 (4.9)	9 (9.1)	0.38
Tricuspid regurtigation > grade 2			
- After 3 months	1 (1.25)	1 (1.15)	0.74
- After 1 years	2 (2.5)	3 (3.45)	0.87
- >1 year	0	3 (3.45)	0.08
Mitral regurgitation > grade 2			
- 3 months	2 (2.5)	2 (2.3)	0.7
- 1 year	3 (3.75)	4 (4.6)	1
- >1 year	2 (2.5)	4 (4.6)	0.51
Mitral gradient (mmHg)			
- 3 months	2.8 ± 1.4	2.9 ± 1.4	0.44
- 1 year	2.9 ± 1.6	2.9 ± 1.7	0.95
- >1 year	2.5 ± 1.2	3.2 ± 1.6	0.05

## Data Availability

Due to ethical restrictions, the data used in this study is unavailable for public sharing.

## Supplementary Materials

The following supporting information can be downloaded at: https://www.mdpi.com/article/10.3390/jpm13060949/s1, Figure S1: Histogram.
