# Supplementary figures and images for: Propensity Matched Outcomes of Minimally Invasive Mitral Surgery: Does a Heart-Team Approach Eliminate Female Gender as an Independent Risk Factor?
Source: J Pers Med. 2023 Jun 3;13(6):949. doi: 10.3390/jpm13060949 (PMC10305141; doi:10.3390/jpm13060949)

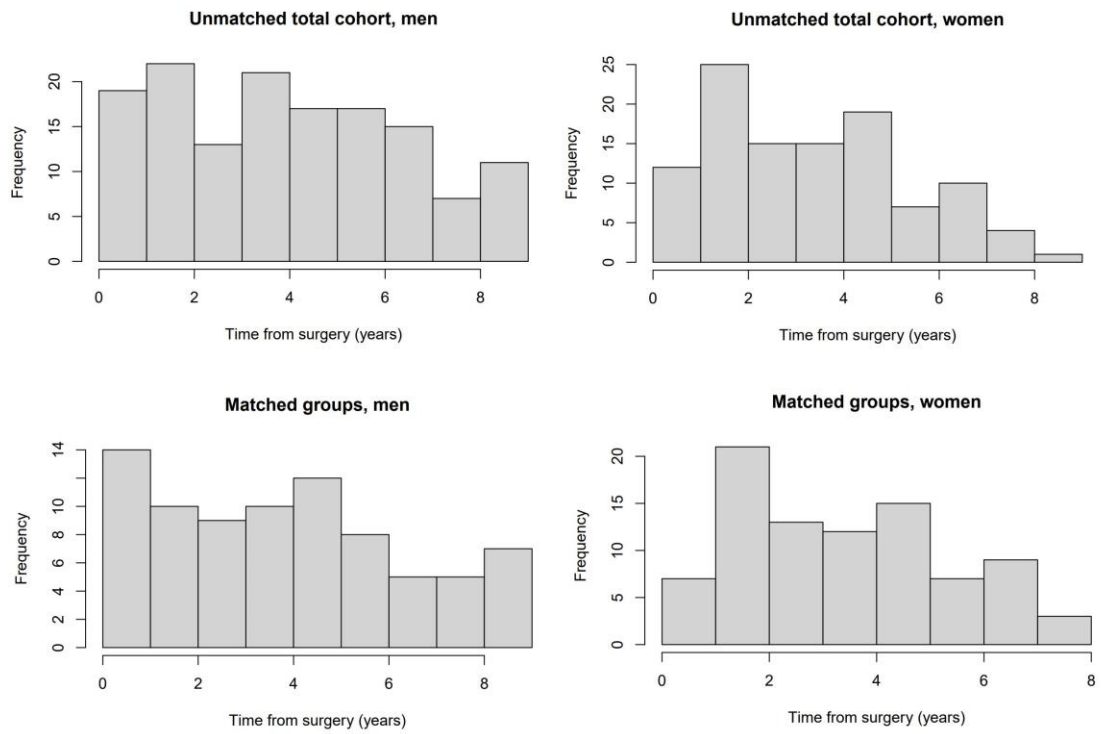

**Figure S1.** Histogram.

Supplement: Supplementary file 1 [file jpm-13-00949-s001.zip › jpm-2342746-Supplementary Materials.pdf]
